# Influence of Translocator Protein (*TSPO*) and rs6971 Variant on Methamphetamine Addiction and Methamphetamine-Associated Psychosis in Turkish Population

**DOI:** 10.3390/life16081344

**Published:** 2026-08-16

**Authors:** Emine Merve Akdağ, Ceren Gümüş, Esra Boztepe, Rukiye Ay Diker, Dilek Pirim

**Affiliations:** 1 Department of Psychiatry, Bursa Yüksek Ihtisas Training and Research Hospital, University of Health Sciences, 16285 Bursa, Türkiye; emervekalyoncu@gmail.com (E.M.A.); rukiye.ay1@saglik.gov.tr (R.A.D.); 2 Department of Translational Medicine, Institute of Health Sciences, Bursa Uludag University, 16059 Bursa, Türkiye; cerengumus1357@gmail.com; 3 Department of Molecular Biology and Genetics, Institute of Natural and Applied Science, Bursa Uludag University, 16059 Bursa, Türkiye; esboztepe99@gmail.com; 4 Department of Molecular Biology and Genetics, Faculty of Arts & Science, Bursa Uludag University, 16210 Bursa, Türkiye

**Keywords:** translocator protein, rs6971, methamphetamine, methamphetamine addiction, methamphetamine associated psychosis, *TSPO*

## Abstract

**Background:** Translocator Protein (TSPO) is a transmembrane protein located in the mitochondrial outer membrane and highly expressed in the brain, which makes it a potential biomarker for microglial activation and neuroinflammation in neurodegenerative diseases. However, its role in neuropsychiatric disorders and its contribution to methamphetamine (METH) addiction (MA) remain unclear. Here, we aimed to investigate the association of the *TSPO* rs6971 with susceptibility to MA and METH-associated psychosis (MAP) and to evaluate its effect on *TSPO* expression. **Methods:** We investigated the association of *TSPO*/rs6971 (Ala147Thr) with susceptibility to MA and METH-associated psychosis (MAP). Genotyping was performed on 300 individuals, including 200 diagnosed with METH use disorder, with 100 of these exhibiting MAP, along with 100 healthy controls (HCs)**.** We explored how different genotypes influence *TSPO* expression at both the transcript and protein levels using RT-qPCR and ELISA. **Results:** Age- and sex-adjusted logistic regression analysis revealed nominal associations between the rs6971 AA genotype and MAP under the recessive and co-dominant genetic models, with stronger associations observed after excluding individuals with a family history of psychiatric disorders. Within the MAP group, carriers of the A allele exhibited increased circulating TSPO levels compared to those with the GG genotype. Additionally, higher protein concentrations (*p* = 0.036) were observed in heterozygous individuals with MAP, compared to METH users without psychosis. **Conclusions:** Our findings suggest that the expression of *TSPO* may be influenced by genotype, with A allele carriers showing elevated *TSPO* expression. Overall, these exploratory findings suggest potential for TSPO-based translational strategies for patients with MA, and highlight the need for more comprehensive evaluations in future research.

## 1. Introduction

Substance use disorders (SUDs) are neuropsychiatric disorders that encompass both substance abuse and substance dependence [1,2]. The etiology of SUDs is influenced by a combination of genetic and environmental factors [3,4,5]. It is estimated that genetic factors account for 40–60% of the heritability of SUDs [1,6].

According to the United Nations Office on Drugs and Crime (UNODC) World Drug Report 2026, amphetamine-type stimulants, particularly methamphetamine (METH), pose a significant public health concern due to the rapidly growing number of users worldwide [7,8]. Despite growing recognition of the public health burden of METH addiction, effective treatment options remain limited [9]. METH users are approximately 11 times more likely to develop psychosis compared to the general population [10]. Moreover, METH-associated psychosis (MAP) accounts for a substantial proportion of patients in psychiatric clinics worldwide [11,12,13,14,15]. Elucidating the molecular risk factors underlying MAP is crucial for improving diagnosis and treatment as the condition places significant strain on healthcare systems, emergency services, and criminal justice systems [16]. Genetic association studies have revealed multiple loci (*SLC1A2*, *GRIA3*, *GRM2*, *HTR2A*, *PACAP*, *BDNF*, and *DISC1*) associated with the risk of developing MAP. However, our knowledge regarding the genetics of METH addiction (MA) and MAP remains limited [13,15,17,18].

TSPO is located on the outer membrane of the mitochondria and is highly expressed in glial cells, endothelial cells, tanycytes, and certain neuronal cells in the brain. It plays an active role in maintaining mitochondrial homeostasis, mitophagy, steroidogenesis, cholesterol transport, as well as neuroinflammation and neuroprotection [19,20]. Therefore, TSPO dysregulations and defects in TSPO have been shown to be associated with various pathological conditions such as Alzheimer’s disease (AD), neurodegenerative diseases, neuroinflammation, brain injury, neuropsychiatric diseases, and cancer [21,22,23,24]. In a recent study, fibroblasts derived from patients with major depressive disorder exhibited reduced *TSPO* and *VDAC1* expression, and *TSPO* deficiency led to reduced mitochondrial membrane potential, impaired calcium homeostasis, increased oxidative stress, and altered mitochondrial dynamics [21]. These findings suggest a potential role for TSPO in depression and emphasize its significance as a therapeutic target for psychiatric disorders due to its impact on cellular energy metabolism and mitochondrial health. While there is substantial evidence supporting the involvement of TSPO in neuropsychiatric diseases, its role in the molecular etiology of METH addiction and MAP remains largely unexplored. This highlights the necessity for further investigation in this area [25].

The rs6971 (G > A) variant is located in the C-terminal domain of the *TSPO* and leads to a change from alanine to threonine at position 147 (Ala147Thr), which affects the structure and stability of the TSPO. Previous studies have also shown that the Ala147Thr significantly alters TSPO’s binding affinity to drug ligands [26]. Since cholesterol also binds to TSPO in the same transmembrane region, it is suggested that this change may hinder TSPO’s ability to bind or uptake cholesterol, thereby impacting steroid synthesis and hypothalamic–pituitary–adrenal function. Moreover, the Ala147Thr change was reported to be associated with anxiety in adults with depression [27]. There is additional evidence indicating that the rs6971/A allele may play a role in the development of bipolar disorder (BD) and modulate the susceptibility to the disease [28,29]. Furthermore, the rs6971 polymorphism was also found to be associated with the development of aggression and impulsivity in adulthood across both genders [30]. A previous study also reported that individuals with the rs6971/AA genotype who had alcohol use disorder experienced more severe withdrawal symptoms compared to those with the GA and GG genotypes [31]. This study noted that the rs6971/AA genotype was associated with higher levels of total cholesterol, LDL cholesterol, and triglycerides. Despite extensive research on this topic, there are currently no studies that have explored the association between this variant and METH addiction in the existing literature. In this study, we aimed to investigate the association between the functional rs6971 variant and METH addiction, as well as MAP. We also sought to assess the effect of individuals’ genotypes on *TSPO* expression.

## 2. Materials and Methods

### 2.1. Samples

Our study involved patients who were admitted to the Psychiatry Department at the Health Sciences University Bursa Yüksek İhtisas Training and Research Hospital and diagnosed with Methamphetamine Use Disorder (MUD) according to the Diagnostic and Statistical Manual of Mental Disorders, 5th edition (DSM-5) [32]. The sample consists of 100 patients with MA, 100 patients with MAP, and 100 healthy controls (HC), all aged between 18 and 65 years. Samples in the HC group had no personal or family history of substance dependence and any psychiatric disorders. The study was conducted in accordance with the ethical guidelines of the Declaration of Helsinki and the research protocol received approval from the ethical committee of the Bursa Yuksek Ihtisas Training and Research Hospital (approval number: 2011-KAEK-25 2023/05-07). Written informed consent was obtained from all participants. The Addiction Profile Index (API) was used to assess the severity and various dimensions of addiction. In parallel, the Brief Psychiatric Rating Scale (BPRS) and Positive and Negative Symptom Scale (PANSS) were employed to evaluate general psychopathology and the intensity of psychotic symptoms among patient volunteers [33,34,35,36]. We determined the total sample size based on a priori power analysis using the pwr package in R software (version 4.4.1). Our sample size achieves 80% power at an alpha level of 0.05, allowing us to detect genetic associations for variants with a minor allele frequency (MAF) value between 0.2 and 0.3, and moderate-to-large genetic effects, corresponding to detectable odds ratios (ORs) of approximately 2 or greater under the dominant model and 4 or greater under the recessive model. The characteristics of the study samples are shown in Table 1.

### 2.2. DNA Isolation

Genomic DNA was isolated from peripheral blood samples collected in EDTA vacutainer tubes. We used PureLink Genomic DNA Mini kit (Thermo Fisher Scientific, Darmstadt, Germany) according to the manufacturer’s instructions. The 260/280 and 260/230 ratios of the DNA samples were assessed using a Nanodrop 2000 spectrophotometer (Thermo Fisher Scientific, Darmstadt, Germany). Additionally, the concentrations of the DNA samples were analyzed with a Qubit fluorometer (Thermo Fisher Scientific, Darmstadt, Germany) using the Qubit dsDNA BR assay (Thermo Fisher Scientific, Darmstadt, Germany).

### 2.3. Genotyping of TSPO/rs6971

Genotyping was performed using a validated TaqMan SNP Genotyping Assay for rs6971 (assay ID: C___2512465_20) and TaqMan Genotyping reagents (Thermo Fisher Scientific, Darmstadt, Germany). A final reaction volume of 10 μL was prepared, consisting of 4.75 μL of genomic DNA (10 ng/μL), 5 μL of 2X TaqMan Genotyping Master Mix, and 0.25 μL of a 40x SNP assay containing VIC/FAM-labeled probes. The PCR protocol was carried out according to manufacturer’s instructions using the QuantGene 9600 Real-Time PCR system (Bioer Technology, Hangzhou, China).

### 2.4. Total RNA Isolation

Total RNA was isolated from whole blood (200 μL) using the miRNeasy Serum/Plasma Advanced Kit (QIAGEN GmbH, Hilden, Germany) following the manufacturer’s protocol. The quantity and quality of the RNA were evaluated using a NanoDrop 2000 spectrophotometer (Thermo Fisher Scientific, Darmstadt, Germany) and Qubit 4 Fluorometer (Thermo Fisher Scientific, Waltham, MA, USA) with the Qubit RNA Assay kit (Thermo Fisher Scientific, Darmstadt, Germany).

### 2.5. Analysis of TSPO Expression by RT-qPCR

Following genotyping, a total of 90 samples were selected from the study cohort for *TSPO* expression analysis. This included 29 samples from the MA group, 33 from the MAP group, and 28 from the HC group. The characteristics of the sample included in gene expression analysis are presented in Supplementary Table S1. cDNA was synthesized using the RevertAid First Strand cDNA Synthesis Kit (Thermo Fisher Scientific, Darmstadt, Germany) from 2 μL of total RNA. The PCR conditions and protocols for cDNA synthesis are detailed in Supplementary Tables S2 and S3. Quantitative PCR (qPCR) analysis was conducted using gene-specific primers for *TSPO* and *β-actin*, as listed in Supplementary Table S4, along with the QuantiNova SYBR Green PCR Kit (QIAGEN GmbH, Hilden, Germany) following the manufacturer’s recommendations. Samples that did not yield a detectable CT value (threshold cycle) in the qPCR analysis were assigned a CT value of 40, representing the maximum cycle number, in accordance with standard practice for non-amplifying samples. This approach was applied to 20 of 29 MA samples, 25 of 33 MAP samples, and 5 of 28 HC samples. However, to evaluate the potential impact of this approach, we also conducted an additional analysis excluding samples with undetermined amplification.

### 2.6. Evaluation of Plasma TSPO Levels by ELISA

Circulating TSPO concentrations were measured using the Human TSPO (Translocator Protein) ELISA Kit (Cat. ELK3592) from ELK Biotechnology (Wuhan, China), following the manufacturer’s instructions. The absorbance of the samples was determined at 490 nm with a BioTek Epoch 2 Microplate Spectrophotometer (Agilent Technologies, Santa Clara, CA, USA). The detectable range for human TSPO was 0.32 and 20 ng/mL, with a sensitivity of 0.109 ng/mL. The intra-assay coefficient of variation was less than 8%, while the inter-assay coefficient of variation was less than 10%.

### 2.7. Statistical and Bioinformatics Analysis

Allele frequencies and Hardy–Weinberg equilibrium (HWE) were calculated by the Haploview software (version 4.2) [37]. The distributions of allele and genotype frequencies were analyzed using chi-square or Fisher’s exact tests. Logistic regression analyses were performed to assess the association between the *TSPO* genotypes and the disease risk. For continuous variables, the normality of data was assessed with the Kolmogorov–Smirnov and Shapiro–Wilk tests. Group comparisons were examined using the non-parametric Mann–Whitney U test, unpaired *t*-test, Kruskal–Wallis test or ANOVA, depending on the data characteristics. Primary analyses were adjusted for age and sex. Additional sensitivity analyses were performed after further adjustment for smoking status. A *p*-value of <0.05 was considered statistically significant. All statistical tests were conducted using IBM SPSS Statistics version 20.0 and GraphPad Prism software version 8.0.2.

Additionally, the LDlink online tool was used to obtain allele frequencies for rs6971 across various global populations (https://ldlink.nih.gov/?tab=home) (accessed on 31 January 2025). The regulatory impact and expression quantitative trait locus (eQTL) effects of rs6971 were obtained from RegulomeDB and the Genotype-Tissue Expression (GTEx) portal (https://www.gtexportal.org/home/) (accessed on 31 January 2025) [38].

## 3. Results

### 3.1. Distribution of Allele and Genotype Frequencies for the TSPO/rs6971

All participants were successfully genotyped for rs6971, and MAF for rs6971/A in the entire sample (n = 300) was 0.232 (Supplementary Table S5). Our study-wide MAF fell within the global MAF range observed in gnomAD (MAF = 0.292), ExAC (MAF = 0.224), and TOPMed (MAF = 0.238) projects. Data from LDlink indicated an MAF range from 1.9% to 39.9% among populations included in the 1000 Genomes project. The MAF observed in our study cohort showed similarities to that of specific populations, including Gambians from the Western Region of Gambia (22.57%), African Americans from the Southwestern United States (21.31%), and Colombians from Medellín (20.21%) (Supplementary Table S6).

However, the MAF was 0.215 in the MA group, 0.275 in the MAP group, and 0.205 in the HC group (Table 2). The distribution of allele and genotype frequencies between groups was assessed by the χ^2^ test, and no statistically significant difference was observed in the entire sample or in gender-stratified groups (Table 2 and Tables S7–S9).

### 3.2. Logistic Regression Analysis

We also assessed the associations between the genotypes and study groups under different genetic models using logistic regression. In the comparison between the MAP group and the HC group, we found that under the co-dominant model, the AA genotype significantly increased the likelihood of developing METH-associated psychosis compared to the GG genotype (β = 1.19, OR = 3.28, *p* = 0.043). Similarly, under the recessive model, the AA genotype was associated with a higher risk of developing METH-induced psychosis compared to both the GG and GA genotypes (β = 1.14, OR = 3.12, *p* = 0.048) (Table 3). Moreover, our data revealed that the rs6971/AA genotype increases the risk of MAP in METH-addicted individuals under the recessive model, with a marginally significant *p*-value (β = 1.04, OR= 2.84, *p* = 0.056). No statistically significant associations were detected in other genetic models. Additional sensitivity analyses that adjusted for smoking status, along with age and sex, attenuated the observed associations (Supplementary Table S10). However, to explore the potential influence of familial psychiatric risk, we conducted an exploratory post-hoc subgroup analysis restricted to METH users without a family history of psychiatric illness (Table 4). In this subgroup, rs6971/AA was significantly associated with the risk of MAP in the recessive and co-dominant models compared to the MA group (recessive model: β = 1.10, OR = 3.01, *p* = 0.047; co-dominant model: β = 1.12, OR = 3.08, *p* = 0.048) and HCs (recessive model: β = 1.28, OR = 3.59, *p* = 0.029; co-dominant model: β = 1.36, OR = 3.89, *p* = 0.023).

### 3.3. TSPO Expression Analysis with RT-qPCR

**Figure 1:**
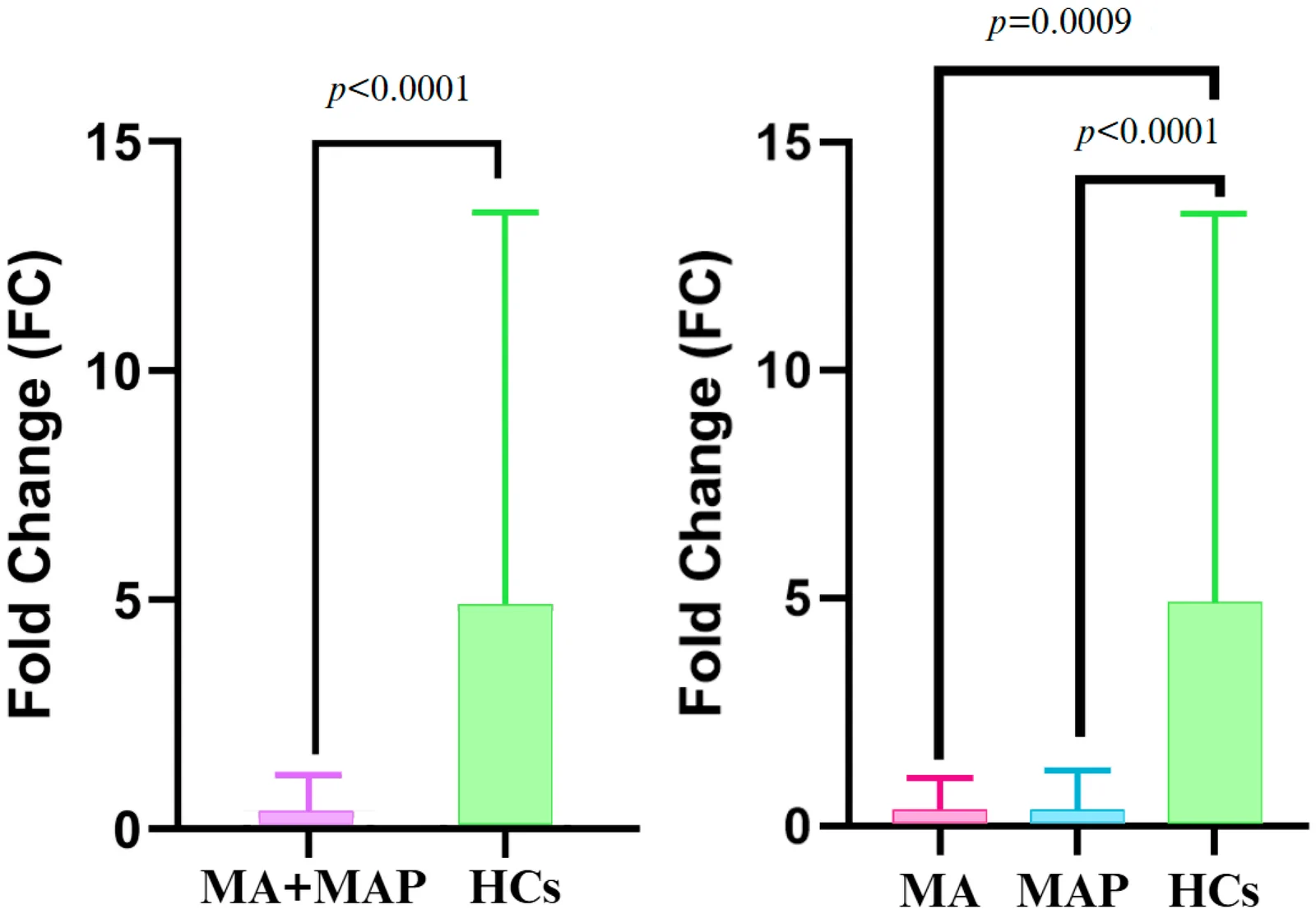
Relative expression levels of *TSPO* in METH users and healthy controls (HCs). The Mann–Whitney U test was used for the two-group comparison, and the Kruskal–Wallis test with Dunn’s post hoc test was used for the three-group comparison.

Our results revealed that individuals with METH-dependence, regardless of the presence of psychosis, exhibit a statistically significant reduction in *TSPO* mRNA levels compared to HCs (Figure 1). The results also remained significant in the sensitivity analysis, yielding significantly lower *TSPO* mRNA expression in METH-dependent groups than in HCs (Mann–Whitney U = 109.5, *p* = 0.018) (Supplementary Figure S1). To assess the impact of genotype on *TSPO* expression, we classified samples with CT<35 as *TSPO* positive (+), while the remaining samples were grouped as *TSPO* negative, as they either showed no detectable levels of peripheral blood *TSPO* mRNA or had levels below the detection threshold of qPCR. Our findings indicate that *TSPO* positivity was significantly higher (*p* = 0.031) in the MA group with the GA genotype compared to those with the GG+AA genotypes, whereas individuals with the GG genotype showed significantly lower (*p* = 0.032) *TSPO* positivity in the MA group (Supplementary Table S11). Given the relatively small subgroup sample size, this finding should be interpreted cautiously and requires confirmation in larger independent cohorts. We did not observe significant differences in the distributions of *TSPO* positivity between the MAP group and HCs.

### 3.4. Concentrations of Plasma TSPO Levels in Relation to rs6971 Genotype

**Figure 2:**
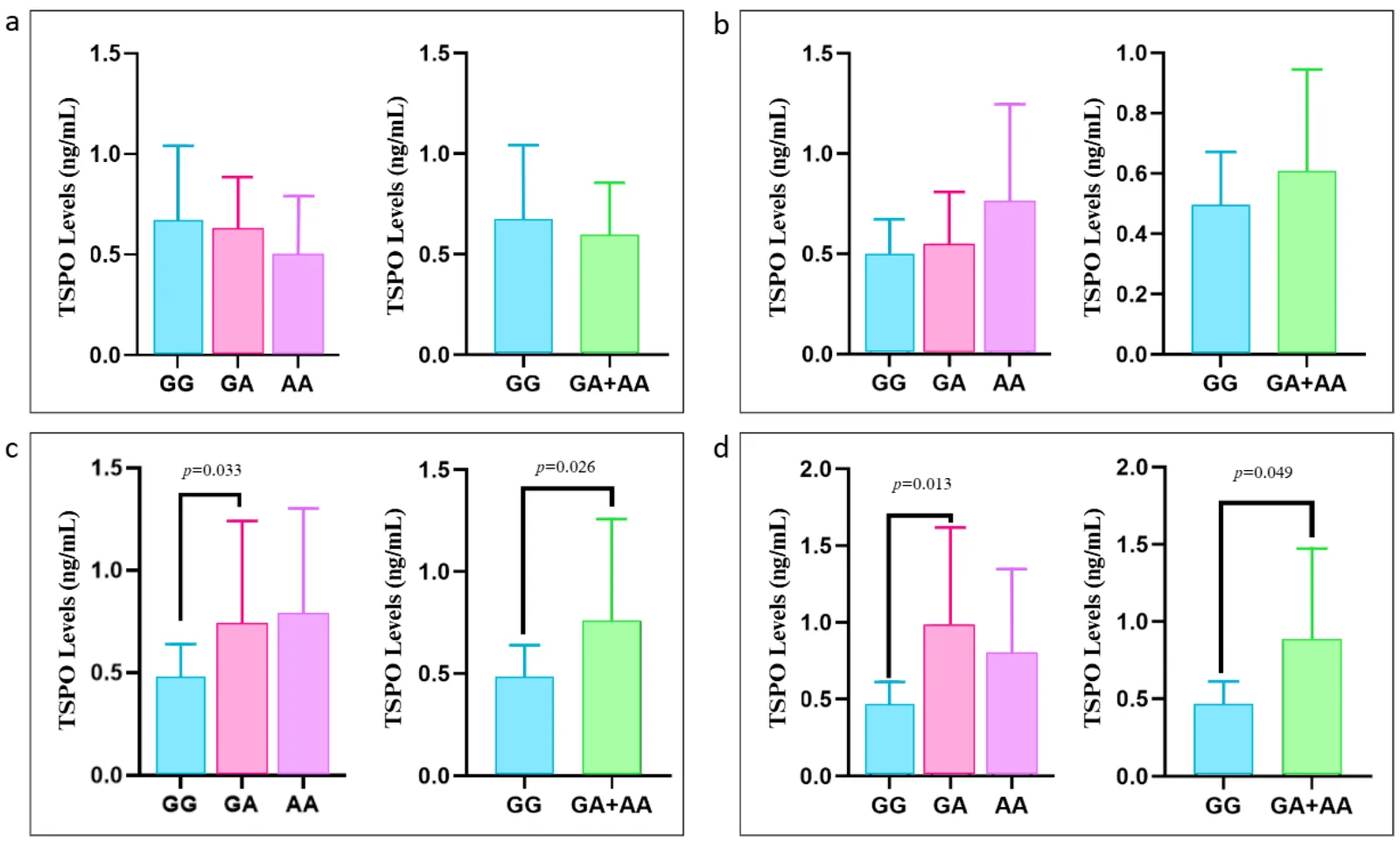
Differences in circulating TSPO levels in (**a**) healthy controls, (**b**) MA, (**c**) MA+MAP and (**d**) MAP grouped by genotypes. The Kruskal–Wallis test with Dunn’s post hoc test was used for GG, GA, and AA comparisons, and the Mann–Whitney U test was used for GG versus GA+AA comparisons.

Plasma TSPO levels were measured successfully in 88 samples, including 28 MA, 32 MAP, and 28 HC samples (Supplementary Table S12). Although no significant difference was observed in plasma TSPO concentrations between the patient and control groups overall, a significant increase (Mann–Whitney U = 31, *p* = 0.036) in TSPO levels was found in heterozygous individuals within the MAP group compared to those in the MA group (Supplementary Table S12). When stratified by genotype, significantly higher TSPO concentrations (Kruskal–Wallis H = 6.287, *p* = 0.043) were detected in carriers of the rs6971/A allele within the MAP group (Figure 2 and Supplementary Table S12). However, no significant correlations were found between plasma TSPO levels and psychopathology scores (PANSS, BPRS, and API).

### 3.5. In-Silico Functional Annotation of TSPO/rs6971

The functional significance of rs6971 was assessed using data from RegulomeDB and the GTEx portal. According to RegulomeDB, rs6971 is assigned a score of 1f, suggesting a possible regulatory role. Data from the GTEx portal revealed that rs6971 is an eQTL that influences the expression of *TSPO* across various tissues, including whole-blood, lung, testis, brain, nerve, breast, and adipose tissue. Notably, in healthy individuals, the major rs6971/GG positively impacts the *TSPO* expression in all tissues, except for the testis. Additionally, the major rs6971/GG negatively influences the expression of *BIK* and *TTLL12* in multiple tissues (Supplementary Table S13).

## 4. Discussion

Prior findings indicate that the *TSPO*/rs6971 causes an amino acid change in TSPO, which affects protein function and interferes with the binding of ligands designed for TSPO tracking in the brain [39]. TSPO is located in the mitochondrial membrane and has multiple roles in mitochondrial and cellular homeostasis, depending on its interactions within molecular networks. Current knowledge emphasizes the potential of TSPO as a neuroinflammation marker, suggesting its use in neuroimaging for neurodegenerative diseases such as AD. However, limited information is available on TSPO’s contribution to the development and progression of psychiatric diseases, and its relevance to SUD remains unclear. Our study provides new insights into the frequency of rs6971 in our population and, for the first time, identifies an association between the minor AA genotype and MAP. Although the frequency of the AA genotype showed only marginal significance when comparing all individuals with MA and MAP, we obtained significant *p*-values when excluding the individuals with a family history of psychiatric diseases in the MA (n = 6) and MAP (n = 17) groups. This suggests that rs6971 may influence MAP risk independently of genetic psychiatric vulnerability. Moreover, API scores were significantly higher in the MA group than in the MAP group, which implies that the severity of METH dependence may not directly correspond to the occurrence of MAP. Further studies are warranted to confirm this finding and clarify the relationship between addiction severity and the risk of psychosis.

We further investigated whether *TSPO* expression is influenced by the rs6971 genotype and analyzed *TSPO* mRNA levels in whole blood using qPCR. RNA sequencing data from healthy individuals in the GTEx database indicate that rs6971 acts as an eQTL in whole blood for *TSPO*; healthy individuals carrying the minor A allele exhibit lower *TSPO* expression than those with the GG genotype. In contrast, we did not find a significant genotype effect on either *TSPO* mRNA expression or circulating TSPO levels in HCs. However, our data suggest that carriers of the A allele may show increased circulating TSPO levels in patients with MAP. This discrepancy may reflect differences in methodology and biological context, as GTEx data are based on *TSPO* mRNA expression in HCs, but our significant finding was observed for plasma TSPO levels in patients with MAP. Moreover, sample size limitations, particularly a limited number of AA carriers, may hinder our ability to detect genotype-dependent effects in HCs. Nevertheless, given the limitation of low *TSPO* expression in whole blood, these findings require further validation using more sensitive methods. Notably, we found that METH users exhibit reduced *TSPO* mRNA levels compared to HCs; however, the difference was not significant when comparing METH users with and without MAP.

Moreover, we also assessed the genotype-dependent changes in circulating TSPO levels in plasma samples from the same individuals analyzed using qPCR. Interestingly, we observed higher protein levels in individuals with MAP who carried the A allele, which suggests a potential functional role of rs6971/A in the development of psychosis among METH users. Previous studies indicate that rs6971 reduces the half-life of the TSPO by 25% and alters its molecular interactions [40,41]. This reduction in protein stability triggers feedback mechanisms that aim to compensate for the rapid degradation of the mutant protein. Additionally, the presence of the A allele could impair TSPO protein function, leading to disruptions in the mitophagy and steroidogenesis pathways, which may exacerbate the effects of METH and contribute to psychosis development. To further understand these findings, future comprehensive analyses should focus on specific cell populations and investigate how genotype affects TSPO and its interacting network.

Furthermore, we observed that circulating TSPO levels did not differ significantly between individuals with METH addiction (MA+MAP) and HCs. Additionally, no significant differences were obtained in TSPO levels between METH users with and without MAP. Previous research indicates that the dysregulation of TSPO levels contributes to disturbances in cellular and mitochondrial functions, influencing specific pathways associated with distinct pathologies. However, findings regarding dysregulation in circulating TSPO levels remain limited [42]. High plasma TSPO concentrations have been found to be associated with poor outcomes in patients with acute ischemic stroke and spontaneous intracerebral hematoma, while individuals with sepsis present low plasma TSPO concentrations [42,43,44]. Moreover, elevated TSPO protein levels were observed in individuals with BD through immunoblotting experiments conducted on PBMCs. These findings suggest that increased TSPO levels may lead to the dysregulation of mitophagy-associated proteins and inhibit the process of mitophagy [45,46].

Our study is the first to evaluate the blood signatures of TSPO using whole-blood samples, which serve as systemic biomarkers and may reflect changes occurring in the brain. Given the invasive nature of blood sampling, exploring biomarker candidates in blood holds significant value for translating findings into clinical practice for mental health disorders [47].

This study has some limitations that need to be acknowledged. First, the inherently low expression of *TSPO* in whole blood may have limited the sensitivity of our analyses to detect genotype-dependent gene expression. Although sensitivity analysis was performed to address the assignment of CT values of 40 to samples with undetermined amplification, the expression results should be interpreted with caution, as they may partly reflect differences in detection rates rather than true expression levels. Further studies using more sensitive techniques, such as digital PCR, are suggested to quantify this effect more precisely. Additionally, while our data show that *TSPO* expression is significantly lower in METH users compared to controls, it is possible that METH use itself may contribute to the suppression of *TSPO* expression, and methodological limitations, including residual confounders, cannot be completely ruled out. Another limitation of the present study is the high prevalence of smoking among individuals with METH use disorder. Although smoking-adjusted sensitivity analyses were performed, the substantial overlap between smoking status and METH use limited our ability to disentangle their independent effects. Therefore, residual confounding by smoking cannot be completely excluded. Similarly, despite adjustment for sex in the regression analyses, the unequal sex distribution across study groups may have resulted in residual confounding that cannot be completely excluded. Further investigation using complementary methodologies such as PET imaging of TSPO or in vitro functional studies is warranted to confirm and extend our findings. In addition, several post hoc subgroup analyses were performed and should therefore be considered exploratory until confirmed in independent cohorts. The deviation from HWE in the MAP group represents another limitation of this study. Although the allelic discrimination plots supported the genotype calls, the findings from the recessive model should be interpreted with caution and independently validated. In addition, sparse data in some subgroup analyses resulted in non-estimable estimates. Although these analyses were considered exploratory, future studies with larger sample sizes and a greater number of AA carriers may yield more stable estimates. While all participants were of Turkish ancestry and were recruited from a large tertiary referral center serving patients from different regions of Türkiye, this single-center study should not be considered representative of the entire Turkish population. Moreover, the relatively small number of individuals with the AA genotype led to wide confidence intervals, which can be considered as another limitation of this study. Finally, given the relatively small sample size, the exploratory nature of the results without multiple-testing correction, and the ethnic specificity of our findings, replication in larger and ethnically diverse populations is essential to confirm the robustness and generalizability.

Nevertheless, our study presents novel and important findings. We identified a significant association between the *TSPO*/rs6971 polymorphism and MAP in a Turkish cohort, marking the first report on this relationship in the literature. Our results highlight a potential genetic vulnerability that may increase the risk of psychosis in METH users and pave the way for future research into TSPO as both a biomarker and a potential therapeutic target. Ultimately, validating this association may support the development of personalized risk assessment tools and TSPO-targeted interventions, offering promising public health benefits for managing substance-induced psychiatric disorders.

## 5. Conclusions

This study investigated the association between the *TSPO*/rs6971 and MA and MAP, together with its potential effect on the *TSPO* gene and protein expression. Our findings provide preliminary evidence suggesting a potential role of rs6971 in susceptibility to MAP and indicate that genotype may influence circulating TSPO levels in individuals with MAP. Given that these associations did not remain significant after multiple-testing correction and should be considered exploratory, further validation in larger, independent cohorts is warranted.

## Figures and Tables

**Table 1:** Demographics and clinical characteristics of the study group.

Characteristics	MA (*n* = 100)	MAP (*n* = 100)	HC (*n* = 100)	*P*_1_	*P*_2_	*P*_3_	*P*_4_	*P*_5_
**Gender *n* F/M**	13/87	20/80	49/51	***p*** ** < 0.001**	***p*** ** < 0.001**	***p*** ** < 0.001**	***p*** ** < 0.001**	0.182
**Age (years)**	31.00 (25.25–36.00)	33.00 (28.00–37.00)	26.50 (22.00–46.00)	**0.030**	0.056	0.401	**0.014**	0.073
**BMI (kg/m^2^)**	24.75 (21.23–27.66)	24.82 (21.82–27.43)	24.82 (22.10–27.89)	0.609	0.456	0.328	0.757	0.515
**Smoking *n* (%)**	99 (99)	99 (99)	44 (44)	***p*** ** < 0.001**	***p*** ** < 0.001**	***p*** ** < 0.001**	***p*** ** < 0.001**	0.999
**Psychiatric family history *n* (%)**	6 (6)	17 (17)	0 (0)	***p*** ** < 0.001**	***p*** ** < 0.001**	**0.029**	***p*** ** < 0.001**	**0.025**
**PANSS Total Score**	-	78.00 (59.25–93.75)	-	-	-	-	-	-
**PANSS Positive**	-	23.67 ± 7.60	-	-	-	-	-	-
**PANSS Negative**	-	14.00 (9.00–18.00)	-	-	-	-	-	-
**PANSS General Psychopathology**	-	40.00 (28.00–51.75)	-	-	-	-	-	-
**BPRS Score**	5.00 (3.00–7.75)	29.00 (19.25–38.75)	-	-	-	-	-	***p*** ** < 0.0001**
**API Score**	12.81 ± 2.53	11.08 ± 2.87	-	-	-	-	-	***p*** ** < 0.0001**
	**MA (*n* = 28)**	**MAP (*n* = 32)**	**HC (*n* = 28)**	***P*** **_1_**	***P*** **_2_**	***P*** **_3_**	***P*** **_4_**	***P*** **_5_**
**TSPO Levels (ng/mL)**	0.41 (0.38–0.70)	0.50 (0.38–1.08)	0.50 (0.38–0.74)	0.641	0.815	0.481	0.805	0.387

Values are reported as mean ± standard deviation (SD) for normally distributed variables and as median (Q1–Q3) for non-normally distributed variables. *P*_1_: Difference between 3 groups; *P*_2_: Difference between the combined MA+MAP group and healthy controls; *P*_3_: Difference between the MA group and healthy controls; *P*_4_: Difference between the MAP group and healthy controls; *P*_5_: Difference between the MA and MAP groups. Statistically significant differences are indicated in bold. HC = Healthy Control; MA = Methamphetamine addiction; MAP = Methamphetamine-associated psychosis. Continuous variables were compared using the Kruskal–Wallis, Mann–Whitney U, or independent-samples *t*-test, as appropriate. Categorical variables were compared using the chi-square or Fisher’s exact test.

**Table 2:** Allele frequency distributions of *TSPO*/rs6971 in the study groups.

	MA+MAP (*n* = 200)	HC (*n* = 100)	χ^2^	*p*
**Alleles**	G:0.755 (75.5%)	G:0.795 (79.5%)	1.199	0.274
	A:0.245 (24.5%)	A:0.205 (20.5%)		
**Genotypes**	GG (*n* = 120)/GA (*n* = 62)/AA (*n* = 18)	GG (*n* = 64)/GA (*n* = 31)/AA (*n* = 5)		
	**MA (*n* = 100)**	**HC (*n* = 100)**		
**Alleles**	G: 0.785 (78.5%)	G: 0.795 (79.5%)	0.060	0.806
	A: 0.215 (21.5%)	A: 0.205 (20.5%)		
**Genotypes**	GG (*n* = 62)/GA (*n* = 33)/AA (*n* = 5)	GG (*n* = 64)/GA (*n* = 31)/AA (*n* = 5)		
	**MAP (*n* = 100)**	**HC (*n* = 100)**		
**Alleles**	G: 0.725 (72.5%)	G: 0.795 (79.5%)	2.686	0.101
	A: 0.275 (27.5%)	A: 0.205 (20.5%)		
**Genotypes**	GG (*n* = 58)/GA (*n* = 29)/AA (*n* = 13)	GG (*n* = 64)/GA (*n* = 31)/AA (*n* = 5)		
	**MA (*n* = 100)**	**MAP (*n* = 100)**		
**Alleles**	G: 0.785 (78.5%)	G: 0.725 (72.5%)	1.946	0.163
	A: 0.215 (21.5%)	A: 0.275 (27.5%)		
**Genotypes**	GG (*n* = 62)/GA (*n* = 33)/AA (*n* = 5)	GG (*n* = 58)/GA (*n* = 29)/AA (*n* = 13)		

HC = Healthy Control; MA = Methamphetamine addiction; MAP = Methamphetamine-associated psychosis. *p* values were calculated using the Chi-square test.

**Table 3:** Logistic regression analysis for the association of *TSPO*/rs6971 genotypes with MA and MAP.

		MA+MAP/HC Total (*n* = 300)					MA+MAP/HC Male (*n* = 218)					MA+MAP/HC Female (*n* = 82)				
		**MA+MAP**	**HC**	**β**	**OR (Cl 95%)**	***p***	**MA+MAP**	**HC**	**β**	**OR (Cl 95%)**	***p***	**MA+MAP**	**HC**	**β**	**OR (Cl 95%)**	***p***
**Gender (M/F)**	-	167/33	51/49	−1.58	0.21 (0.12–0.35)	*p* < 0.001	167	51	-	-	-	33	49	-	-	-
**Age (year)**	-	-	-	-	-	-	-	-	-	-	-	-	-	-	-	-
**Dominant**	AA+GA vs. GG	80–120	36–64	0.31	1.36 (0.80–2.32)	0.258	65–102	15–36	0.43	1.53 (0.78–3.01)	0.219	15–18	21–28	0.11	1.11 (0.46–2.70)	0.816
**Recessive**	AA vs. GA+GG	18–182	5–95	0.69	1.99 (0.67–5.85)	0.214	14–153	3–48	0.38	1.46 (0.40–5.31)	0.562	4–29	2–47	1.18	3.24 (0.56–18.83)	0.190
**Co-dominant**	GG	120	64	-	-	Ref	102	36	-	-	Ref	18	28	-	-	Ref
	GA vs. GG	62–120	31–64	0.21	1.23 (0.70–2.18)	0.468	51–102	12–36	0.41	1.50 (0.72–3.13)	0.279	11–18	19–28	−0.11	0.90 (0.35–2.33)	0.829
	AA vs. GG	18–120	5–64	0.76	2.13 (0.71–6.40)	0.177	14–102	3–36	0.50	1.65 (0.45–6.07)	0.453	4–18	2–28	1.14	3.11 (0.52–18.78)	0.216
**Overdominant**	GA vs. GG+AA	62–138	31–69	0.13	1.14 (0.66–1.99)	0.639	51–116	12–39	0.36	1.43 (0.69–2.95)	0.335	11–22	19–30	−0.24	0.79 (0.31–1.99)	0.616
**Additive**	-	-	-	0.30	1.35 (0.89–2.06)	0.163	-	-	0.32	1.38 (0.81–2.34)	0.239	-	-	0.27	1.31 (0.65–2.62)	0.454
		**MA/HC Total (*n* = 200)**					**MA/HC Male (*n* = 138)**					**MA/HC Female (*n* = 62)**				
		**MA**	**HC**	**β**	**OR (Cl 95%)**	***p***	**MA**	**HC**	**β**	**OR (Cl 95%)**	***p***	**MA**	**HC**	**β**	**OR (Cl 95%)**	***p***
**Gender (M/F)**	-	87/13	51/49	−1.86	0.16 (0.08–0.31)	*p* < 0.001	87	51	-	-	-	13	49	-	-	-
**Age (year)**	-	-	-	-	-	-	-	-	-	-	-	-	-	-	-	-
**Dominant**	AA+GA vs. GG	38–62	36–64	0.28	1.32 (0.70–2.50)	0.386	32–55	15–36	0.33	1.40 (0.66–2.94)	0.379	6–7	21–28	0.13	1.14 (0.34–3.90)	0.831
**Recessive**	AA vs. GA+GG	5–95	5–95	−0.22	0.80 (0.21–3.09)	0.748	5–82	3–48	−0.03	0.98 (0.22–4.26)	0.974	0–13	2–47	NE	NE	-
**Co-dominant**	GG	62	64	-	-	Ref	55	36	-	-	Ref	7	28	-	-	Ref
	GA vs. GG	33–62	31–64	0.11	1.12 (0.29–4.39)	0.872	27–55	12–36	0.39	1.47 (0.66–3.28)	0.343	6–7	19–28	0.23	1.26 (0.37–4.35)	0.711
	AA vs. GG	5–62	5–64	0.46	1.59 (0.38–6.64)	0.527	5–55	3–36	0.09	1.09 (0.25–4.85)	0.909	0–7	2–28	NE	NE	-
**Overdominant**	GA vs. GG+AA	33–67	31–69	0.36	1.43 (0.74–2.78)	0.291	27–60	12–39	0.38	1.46 (0.66–3.22)	0.346	6–7	19–30	0.30	1.35 (0.40–4.64)	0.630
**Additive**	-	-	-	0.16	1.17 (0.70–1.96)	0.558	-	-	0.21	1.23 (0.68–2.23)	0.492	-	-	−0.03	0.98 (0.33–2.92)	0.964
		**MAP/HC Total (*n* = 200)**					**MAP/HC Male (*n* = 131)**					**MAP/HC Female (*n* = 69)**				
		**MAP**	**HC**	**β**	**OR (Cl 95%)**	***p***	**MAP**	**HC**	**β**	**OR (Cl 95%)**	***p***	**MAP**	**HC**	**β**	**OR (Cl 95%)**	***p***
**Gender (M/F)**	-	80/20	51/49	−1.35	0.26 (0.14–0.49)	*p* < 0.001	80	51	-	-	-	20	49	-	-	-
**Age (year)**	-	-	-	0.00	1.00 (0.98–1.03)	0.990	-	-	0.00	1.00 (0.97–1.04)	0.823	-	-	-	-	-
**Dominant**	AA+GA vs. GG	42–58	36–64	0.38	1.46 (0.80–2.67)	0.223	33–47	15–36	0.55	1.73 (0.81–3.71)	0.156	9–11	21–28	0.09	1.09 (0.38–3.11)	0.871
**Recessive**	AA vs. GA+GG	13–87	5–95	1.14	3.12 (1.01–9.63)	**0.048**	9–71	3–48	0.71	2.04 (0.52–7.92)	0.304	4–16	2–47	1.77	5.88 (0.98–35.18)	0.052
**Co-dominant**	GG	58	64	-	-	Ref	47	36	**-**	-	Ref	11	28	-	-	Ref
	GA vs. GG	29–58	31–64	0.15	1.17 (0.60–2.25)	0.649	24–47	12–36	0.46	1.58 (0.69–3.62)	0.281	5–11	19–28	−0.40	0.67 (0.20–2.24)	0.515
	AA vs. GG	13–58	5–64	1.19	3.28 (1.04–10.38)	**0.043**	9–47	3–36	0.85	2.34 (0.59–9.29)	0.228	4–11	2–28	1.63	5.09 (0.81–31.90)	0.082
**Overdominant**	GA vs. GG+AA	29–71	31–69	0.01	1.01 (0.53–1.91)	0.985	24–56	12–39	0.35	1.43 (0.63–3.22)	0.394	5–15	19–30	−0.64	0.53 (0.16–1.69)	0.280
**Additive**	-	-	-	0.42	1.52 (0.96–2.41)	0.075	-	-	0.44	1.55 (0.87–2.75)	0.137	-	-	0.41	1.51 (0.69–3.30)	0.300
		**MAP/MA Total (*n* = 200)**					**MAP/MA Male (*n* = 167)**					**MAP/MA Female (*n* = 33)**				
		**MAP**	**MA**	**β**	**OR (Cl 95%)**	***p***	**MAP**	**MA**	**β**	**OR (Cl 95%)**	***p***	**MAP**	**MA**	**β**	**OR (Cl 95%)**	***p***
**Gender (M/F)**	-	80/20	87/13	-	-	-	80	87	-	-	-	20	13	-	-	-
**Age (year)**	-	-	-	-	-	-	-	-	-	-	-	-	-	-	-	-
**Dominant**	AA+GA vs. GG	42–58	38–62	0.17	1.18 (0.67–2.08)	0.564	33–47	32–55	0.19	1.21 (0.65–2.25)	0.554	9–11	6–7	−0.05	0.96 (0.24–3.88)	0.948
**Recessive**	AA vs. GA+GG	13–87	5–95	1.04	2.84 (0.97–8.29)	0.056	9–71	5–82	0.73	2.08 (0.67–6.49)	0.208	4–16	0–13	NE	NE	-
**Co-dominant**	GG	58	62	-	-	Ref	47	55	-	-	Ref	11	7	-	-	Ref
	GA vs. GG	29–58	33–62	−0.06	0.94 (0.51–1.74)	0.842	24–47	27–55	0.04	1.04 (0.53–2.04)	0.909	5–11	6–7	−0.63	0.53 (0.12–2.42)	0.413
	AA vs. GG	13–58	5–62	1.02	2.78 (0.93–8.28)	0.066	9–47	5–55	0.75	2.11 (0.66–6.72)	0.208	4–11	0–7	NE	NE	-
**Overdominant**	GA vs. GG+AA	29–71	33–67	−0.19	0.83 (0.46–1.51)	0.541	24–56	27–60	−0.05	0.95 (0.49–1.84)	0.885	5–15	6–7	−0.94	0.39 (0.09–1.72)	0.213
**Additive**	-	-	-	0.28	1.33 (0.86–2.03)	0.197	-	-	0.24	1.27 (0.79–2.04)	0.320	-	-	0.40	1.50 (0.52–4.27)	0.452

Associations were assessed using binary logistic regression. In the dominant and co-dominant models, the GG genotype was used as the reference group; in the recessive model, GA+GG was used as the reference group; and in the overdominant model, GG+AA was used as the reference group. Male was used as the reference category for gender. Age and gender were included as covariates in the relevant models. None of the nominal associations remained statistically significant after Benjamini–Hochberg FDR correction (all q values > 0.05). Nominal *p* values < 0.05 are presented in bold. β, beta coefficient; OR, odds ratio; CI, confidence interval; *n*, number of individuals; HC, healthy control; MA, Methamphetamine addiction; MAP, methamphetamine-associated psychosis; NE, not estimable due to zero cell counts and complete separation.

**Table 4:** Associations of *TSPO*/rs6971 genotypes with MAP in individuals with no family history of psychiatric illness.

		MAP/HC Total (*n* = 183)				
		**MAP (*n* = 83)**	**HC (*n* = 100)**	**β**	**OR (Cl 95%)**	***p***
**Gender (M/F)**	-	66/17	51/49	−1.36	0.26 (0.13–0.51)	*p* < 0.001
**Age (year)**	-	-	-	−0.00	1.00 (0.97–1.03)	0.932
**Recessive**	AA vs. GA+GG	12–71	5–95	1.28	3.59 (1.14–11.31)	**0.029**
**Co-dominant**	GG	46	64	-	-	Ref
	GA vs. GG	25–46	31–64	0.23	1.26 (0.63–2.50)	0.510
	AA vs. GG	12–46	5–64	1.36	3.89 (1.20–12.60)	**0.023**
		**MAP/MA Total (*n* = 177)**				
		**MAP (*n* = 83)**	**MA (*n* = 94)**	**β**	**OR (Cl 95%)**	***p***
**Gender (M/F)**	-	66/17	82/12			
**Age (year)**	-	-	-	-	-	-
**Recessive**	AA vs. GA+GG	12–71	5–84	1.10	3.01 (1.01–8.94)	**0.047**
**Co-dominant**	GG	46	30	-	-	Ref
	GA vs. GG	25–46	30–54	0.07	1.07 (0.56–2.06)	0.842
	AA vs. GG	12–46	5–54	1.12	3.08 (1.01–9.36)	**0.048**

Associations were assessed using binary logistic regression. In the co-dominant model, the GG genotype was used as the reference group; for the recessive model, the GA+GG genotypes were used as the reference; Male was selected as the reference group for gender. Age and gender were included in the model as covariates; β: Beta coefficient; OR: Odds ratio; CI: Confidence interval; *n*: Number of individuals. Statistically significant results are presented in bold. HC = Healthy control; MA = Methamphetamine addiction; MAP = Methamphetamine-associated psychosis.

## Data Availability

The data presented in this study are available on request from the corresponding author.

## Supplementary Materials

The following supporting information can be downloaded at https://www.mdpi.com/article/10.3390/life16081344/s1: Supplementary Table S1. Sample characteristics included in gene expression; Supplementary Table S2. Reagents used for cDNA synthesis; Supplementary Table S3. PCR conditions used for cDNA synthesis; Supplementary Table S4. Primer sequences used for qRT-PCR; Supplementary Table S5. Allele and genotype frequencies of *TSPO*/rs6971 in the entire sample (*n* = 300); Supplementary Table S6. Allele frequency distributions of rs6971 in worldwide populations (Available online: https://ldlink.nih.gov (accessed on 31 January 2025)); Supplementary Table S7. Genotype distributions of rs6971 in individuals with METH dependence and healthy controls; Supplementary Table S8. Allele frequency distributions of *TSPO*/rs6971 in females; Supplementary Table S9. Allele frequency distributions of *TSPO*/rs6971 in males; Supplementary Table S10. Smoking-adjusted sensitivity logistic regression analyses of the association between *TSPO* rs6971 genotypes and MA/MAP status; Supplementary Table S11. Distribution of *TSPO*/rs6971 genotypes in *TSPO* (+) and *TSPO* (-) samples determined by qRT-PCR; Supplementary Table S12. Comparison of TSPO protein levels in the study groups stratified by genotype; Supplementary Table S13. Functional relevance of rs6971 retrieved from RegulomeDB and GTEx databases; Supplementary Figure S1. Analysis of relative *TSPO* expression levels following the exclusion of samples assigned a Ct value of 40. The Mann–Whitney U test was used for the two-group comparison, and the Kruskal–Wallis test with Dunn’s post hoc test was used for the three-group comparison.

## Abbreviations

The following abbreviations are used in this manuscript:

AD	Alzheimer’s disease
Ala147Thr	Alanine to threonine substitution at amino acid position 147
API	Addiction Profile Index
BD	Bipolar disorder
BPRS	Brief Psychiatric Rating Scale
cDNA	Complementary DNA
DSM-5	Diagnostic and Statistical Manual of Mental Disorders, Fifth Edition
eQTL	Expression quantitative trait locus
ExAC	Exome Aggregation Consortium
GTEx	Genotype-Tissue Expression
HC	Healthy control
HWE	Hardy–Weinberg equilibrium
MA	Methamphetamine addiction
MAF	Minor allele frequency
MAP	Methamphetamine-associated psychosis
METH	Methamphetamine
MUD	Methamphetamine Use Disorder
PANSS	Positive and Negative Syndrome Scale
PCR	Polymerase chain reaction
qPCR	Quantitative polymerase chain reaction
RNA	Ribonucleic acid
SUDs	Substance use disorders
TSPO	Translocator protein

## References

[1] Deak J.D., Johnson E.C. (2021). Genetics of substance use disorders: A review. Psychol. Med..

[2] Volkow N.D., Blanco C. (2023). Substance use disorders: A comprehensive update of classification, epidemiology, neurobiology, clinical aspects, treatment and prevention. World Psychiatry.

[3] Gelernter J., Polimanti R. (2021). Genetics of substance use disorders in the era of big data. Nat. Rev. Genet..

[4] Hatoum A.S., Colbert S.M.C., Johnson E.C., Huggett S.B., Deak J.D., Pathak G.A., Jennings M.V., Paul S.E., Karcher N.R., Hansen I. (2023). Multivariate genome-wide association meta-analysis of over 1 million subjects identifies loci underlying multiple substance use disorders. Nat. Ment. Health.

[5] Prom-Wormley E.C., Ebejer J., Dick D.M., Bowers M.S. (2017). The genetic epidemiology of substance use disorder: A review. Drug Alcohol Depend..

[6] Gerring Z.F., Thorp J.G., Treur J.L., Verweij K.J.H., Derks E.M. (2024). The genetic landscape of substance use disorders. Mol. Psychiatry.

[7] Guerin A.A., Nestler E.J., Berk M., Lawrence A.J., Rossell S.L., Kim J.H. (2021). Genetics of methamphetamine use disorder: A systematic review and meta-analyses of gene association studies. Neurosci. Biobehav. Rev..

[8] United Nations Office on Drugs and Crime (UNODC) (2026). World Drug Report 2026.

[9] Khalili M., Sadeghirad B., Bach P., Crabtree A., Javadi S., Sadeghi E., Moradi S., Fashami F.M., Nakhaeizadeh M., Salehi S. (2025). Management of amphetamine and methamphetamine use disorders: A systematic review and network meta-analysis of randomized trials. Int. J. Ment. Health Addict..

[10] McKetin R., McLaren J., Lubman D.I., Hides L. (2006). The prevalence of psychotic symptoms among methamphetamine users. Addiction.

[11] Barr A.M., Panenka W.J., MacEwan G.W., Thornton A.E., Lang D.J., Honer W.G., Lecomte T. (2006). The need for speed: An update on methamphetamine addiction. J. Psychiatry Neurosci..

[12] Kishimoto M., Ujike H., Motohashi Y., Tanaka Y., Okahisa Y., Kotaka T., Harano M., Inada T., Yamada M., Komiyama T. (2008). The dysbindin gene (DTNBP1) is associated with methamphetamine psychosis. Biol. Psychiatry.

[13] Turan Ç., Şenormancı G., Şenormancı Ö., Çelik S.K., Çakmak G., Demirci O.O. (2023). Comparison of pituitary adenylate cyclase-activating polypeptide (PACAP, ADCYAP1) gene polymorphisms among patients with methamphetamine addiction, methamphetamine-induced psychosis and healthy controls. Ann. Méd.-Psychol. Rev. Psychiatr..

[14] Wearne T.A., Cornish J.L. (2018). A Comparison of Methamphetamine-Induced Psychosis and Schizophrenia: A Review of Positive, Negative, and Cognitive Symptomatology. Front. Psychiatry.

[15] Yahya D.N., Mac Guad R., Wu Y.-S., Gan S.H., Gopinath S.C.B., Zakariah H.A., Rashid R.A., Sim M.S. (2023). SLC1A2 Gene Polymorphism Influences Methamphetamine-Induced Psychosis. J. Pers. Med..

[16] Stacy P., Frantz J., Miller G., Merrill B., Gainer D. (2024). A Narrative Review of the Pathophysiology and Treatment of Methamphetamine-Associated Psychosis. Int. J. Ment. Heal. Addict..

[17] Iamjan S.-A., Thanoi S., Watiktinkorn P., Reynolds G.P., Nudmamud-Thanoi S. (2018). Genetic variation of GRIA3 gene is associated with vulnerability to methamphetamine dependence and its associated psychosis. J. Psychopharmacol..

[18] Tsunoka T., Kishi T., Kitajima T., Okochi T., Okumura T., Yamanouchi Y., Kinoshita Y., Kawashima K., Naitoh H., Inada T. (2010). Association analysis of GRM2 and HTR2A with methamphetamine-induced psychosis and schizophrenia in the Japanese population. Prog. Neuro-Psychopharmacol. Biol. Psychiatry.

[19] Bilgin E.E., Pirim D., Soydan G. (2024). A *p*− adic approach to the TSPO gene. BioSystems.

[20] El Chemali L., Akwa Y., Massaad-Massade L. (2022). The mitochondrial translocator protein (TSPO): A key multifunctional molecule in the nervous system. Biochem. J..

[21] Bader S., Jahner T., Dörfelt A., Melchner D., Cardon I., Siegmund H.I., Brochhausen C., Rupprecht R., Milenkovic V.M., Wetzel C.H. (2024). A Comprehensive Functional Investigation of the Human Translocator Protein 18 kDa (TSPO) in a Novel Human Neuronal Cell Knockout Model. Int. J. Mol. Sci..

[22] Luu T.G., Kim H.-K. (2022). 18F-Radiolabeled Translocator Protein (TSPO) PET Tracers: Recent Development of TSPO Radioligands and Their Application to PET Study. Pharmaceutics.

[23] Simpson D., Gharehgazlou A., Da Silva T., Labrie-Cleary C., Wilson A.A., Meyer J.H., Mizrahi R., Rusjan P.M. (2022). In vivo imaging translocator protein (TSPO) in autism spectrum disorder. Neuropsychopharmacology.

[24] Tournier B.B., Tsartsalis S., Ceyzériat K., Garibotto V., Millet P. (2020). In Vivo TSPO Signal and Neuroinflammation in Alzheimer’s Disease. Cells.

[25] Elma Y., Yılmaz Can E. (2024). TSPO’nun (18 kDa Translokatör Protein) Yapısı, İşlevi ve Patolojik Süreçlerdeki Rolü. Med. J. West. Black Sea.

[26] Salerno S., Viviano M., Baglini E., Poggetti V., Giorgini D., Castagnoli J., Barresi E., Castellano S., Da Settimo F., Taliani S. (2024). TSPO Radioligands for Neuroinflammation: An Overview. Molecules.

[27] Costa B., Pini S., Martini C., Abelli M., Gabelloni P., Landi S., Muti M., Gesi C., Lari L., Cardini A. (2009). Ala147Thr substitution in translocator protein is associated with adult separation anxiety in patients with depression. Psychiatr. Genet..

[28] Colasanti A., Owen D.R., Grozeva D., Rabiner E.A., Matthews P.M., Craddock N., Young A.H. (2013). Bipolar Disorder is associated with the rs6971 polymorphism in the gene encoding 18 kDa Translocator Protein (TSPO). Psychoneuroendocrinology.

[29] Prossin A.R., Chandler M., Ryan K.A., Saunders E.F., Kamali M., Papadopoulos V., Zöllner S., Dantzer R., McInnis M.G. (2018). Functional TSPO polymorphism predicts variance in the diurnal cortisol rhythm in Bipolar Disorder. Psychoneuroendocrinology.

[30] Vaht M. (2021). Variation rs6971 in the Translocator Protein Gene (TSPO) is Associated with Aggressiveness and Impulsivity but Not with Anxiety in a Population-Representative Sample of Young Adults. J. Genet. Psychol..

[31] Wiers C.E., De Carvalho L.M., Hodgkinson C.A., Schwandt M., Kim S.W., Diazgranados N., Wang G., Goldman D., Volkow N.D. (2021). TSPO polymorphism in individuals with alcohol use disorder: Association with cholesterol levels and withdrawal severity. Addict. Biol..

[32] Hasin D.S., O’brien C.P., Auriacombe M., Borges G., Bucholz K., Budney A., Compton W.M., Crowley T., Ling W., Petry N.M. (2013). DSM-5 Criteria for Substance Use Disorders: Recommendations and Rationale. Am. J. Psychiatry.

[33] Hofmann A.B., Schmid H.M., Jabat M., Brackmann N., Noboa V., Bobes J., Garcia-Portilla M.P., Seifritz E., Vetter S., Egger S.T. (2022). Utility and validity of the Brief Psychiatric Rating Scale (BPRS) as a transdiagnostic scale. Psychiatry Res..

[34] Kostakoğlu E., Batur S., Tiryaki A., Göğüş A. (1999). Pozitif ve Negatif Sendrom Ölçeğinin (PANSS) Türkçe uyarlamasının geçerlilik ve güvenilirliği. Türk Psikol. Derg..

[35] Overall J.E., Gorham D.R. (1962). The Brief Psychiatric Rating Scale. Psychol. Rep..

[36] Ögel K., Evren C., Gürol D.T., Karadağ F. (2012). Bağımlılık Profil İndeksi’nin (BAPİ) geliştirilmesi, geçerlik ve güvenilirliği. Türk Psikiyatr. Derg..

[37] Barrett J.C., Fry B., Maller J., Daly M.J. (2005). Haploview: Analysis and visualization of LD and haplotype maps. Bioinformatics.

[38] Boyle A.P., Hong E.L., Hariharan M., Cheng Y., Schaub M.A., Kasowski M., Karczewski K.J., Park J., Hitz B.C., Weng S. (2012). Annotation of functional variation in personal genomes using RegulomeDB. Genome Res..

[39] Berroterán-Infante N., Tadić M., Hacker M., Wadsak W., Mitterhauser M. (2019). Binding Affinity of Some Endogenous and Synthetic TSPO Ligands Regarding the rs6971 Polymorphism. Int. J. Mol. Sci..

[40] Asih P.R., Poljak A., Kassiou M., Ke Y.D., Ittner L.M. (2022). Differential mitochondrial protein interaction profile between human translocator protein and its A147T polymorphism variant. PLoS ONE.

[41] Milenkovic V.M., Bader S., Sudria-Lopez D., Siebert R., Brandl C., Nothdurfter C., Weber B.H.F., Rupprecht R., Wetzel C.H. (2018). Effects of genetic variants in the TSPO gene on protein structure and stability. PLoS ONE.

[42] Hattori M., Kikutani K., Hosokawa K., Kyo M., Nishikimi M., Ota K., Ohshimo S., Aizawa H., Shime N. (2024). Diagnostic utility of plasma translocator protein 18 kDa (TSPO) in sepsis: A case-control study. Medicine.

[43] Chen W.-H., Yeh H.-L., Tsao C.-W., Lien L.-M., Chiwaya A., Alizargar J., Bai C.-H. (2018). Plasma Translocator Protein Levels and Outcomes of Acute Ischemic Stroke: A Pilot Study. Dis. Markers.

[44] Jiang W., Jin P., Bao Q., Wei W., Jiang W. (2021). Prognostic significance of serum translocator protein in patients with spontaneous intracerebral hematoma: Preliminary findings. Neurol. Res..

[45] Matutino Santos P., Pereira Campos G., Nascimento C. (2023). Endo-Lysosomal and Autophagy Pathway and Ubiquitin-Proteasome System in Mood Disorders: A Review Article. Neuropsychiatr. Dis. Treat..

[46] Scaini G., Barichello T., Fries G.R., Kennon E.A., Andrews T., Nix B.R., Zunta-Soares G., Valvassori S.S., Soares J.C., Quevedo J. (2019). TSPO upregulation in bipolar disorder and concomitant downregulation of mitophagic proteins and NLRP3 inflammasome activation. Neuropsychopharmacology.

[47] Hayashi-Takagi A., Vawter M.P., Iwamoto K. (2014). Peripheral Biomarkers Revisited: Integrative Profiling of Peripheral Samples for Psychiatric Research. Biol. Psychiatry.

