# Supplementary material for: Influence of Translocator Protein (TSPO) and rs6971 Variant on Methamphetamine Addiction and Methamphetamine-Associated Psychosis in Turkish Population
Source: Life (Basel). 2026 Aug 16;16(8):1344. doi: 10.3390/life16081344 (PMC13514851; doi:10.3390/life16081344)
Supplement: Supplementary file 1 [file life-16-01344-s001.zip › life-4423137-supplementary.pdf]

SUPPLEMENTARY TABLES

Supplementary Table S1. Sample characteristics included in gene expression.

		MA (n=29)	MAP (n=33)	HC (n=28)	P_1	P_2	P_3	P_4	P_5
**Year**		31.00 (25.00–36.00)	32.00 (27.50–37.00)	25.00 (22.25–43.00)	0.188	0.196	0.609	0.092	0.116
**Gender**	Male n (%)	23 (25.59)	24 (26.67)	17 (18.89)	0.292	0.144	0.125	0.319	0.546
	Female n (%)	6 (6.67)	9 (10.00)	11 (12.22)					
**Genotype**	GG n (%)	10 (11.11)	11 (12.22)	11 (12.22)	0.514	0.701	0.912	0.387	0.300
	GA n (%)	14 (15.56)	11 (12.22)	12 (13.33)					
	AA n (%)	5 (5.56)	11 (12.22)	5 (5.56)					

Values are reported as median (Q1–Q3). P_1: Difference between 3 groups; P_2: Difference between the combined MA+MAP group and healthy controls; P_3: Difference between the MA group and healthy controls; P_4: Difference between the MAP group and healthy controls; P_5: Difference between the MA and MAP groups. Statistically significant differences are indicated in bold. HC=Healthy Controls; MA= Methamphetamine addiction; MAP=Methamphetamine-associated psychosis. Age was compared using the Kruskal–Wallis or Mann–Whitney U test. Gender and genotype distributions were compared using the chi-square or Fisher's exact test.

Supplementary Table S2. Reagents used for cDNA synthesis.

	Volume
**RNA**	2ul
**Primer (Random Hexomer)**	1ul
**Water**	9ul
**5x Reaction Buffer**	4ul
**RNA Inhibitor**	1ul
**10mM dNTP Mix**	2ul
**Revetaid M-MuLV RT**	1ul
**Total Volume**	20ul

Supplementary Table S3. PCR conditions used for cDNA synthesis.

	Time	Temperature
**Reverse Transcription**	60 min.	42°C
	5 min.	25°C
**Inactivation**	5 min.	70°C

Supplementary Table S4. Primer sequences used for qRT-PCR.

Gene	Primer	Sequences (5'-> 3')	Amplicon Length (bp)
TSPO	Forward	TTCACAGAGAAGGCTGTGGTTC	247
	Reverse	GCCATACGCAGTAGTTGAGTGT	
β-actin	Forward	TTAATAGTCATTCCAAATATGA	246
	Reverse	GGGACAAAAAAGGGGGAAGG	

Supplementary Table S5. Allele and genotype frequencies of *TSPO*/rs6971 in the entire sample (n=300).

RefSNP ID	Chr loc.[a]	HWE-*p*[b]	Genotype (n, %)		1000G[c]	gnomAD v4 Exomes[d]	ExAC[e]	TOPMed[f]
**rs6971**	43162920		**Entire sample**		0.172 (n= 5008)	0.292 (n= 1381372)	0.224 (n= 81568)	0.238 (n= 264690)
		0.039	GG (184, 61.33)	**MAF**=0.232				
			GA (93, 31.00)					
			AA (23, 7.67)					
			**MA**					
		0.991	GG (62, 62.00)	**MAF**=0.215				
			GA (33, 33.00)					
			AA (5, 5.00)					
			**MAP**					
		0.013	GG (58, 58.00)	**MAF**=0.275				
			GA (29, 29.00)					
			AA (13, 13.00)					
			**HC**					
		0.788	GG (64, 64.00)	**MAF**=0.205				
			GA (31, 31.00)					
			AA (5, 5.00)					

a; Chromosomal location on chromosome 22 (GRCh38.p14), b; Hardy-Weinberg P value, c; 1000 Genome Project MAF in global, d; The Genome Aggregation Database v4 Exomes MAF in global, e; The Exome Aggregation Consortium MAF in global, f; Trans-Omics for Precision Medicine (TOPMed) MAF in global. HC= Healthy Controls; MA= Methamphetamine addiction; MAP=Methamphetamine-associated psychosis; MAF= minor allele frequency. HWE p values were calculated using Haploview 4.2.

Supplementary Table S6. Allele frequency distributions of rs6971 in worldwide populations (retrieved from https://ldlink.nih.gov).

Population	Abbreviation	N	rs6971 Allele Freq
**Turkish Populations**	-	300	A: 23.2%, G: 76.8%
**All Populations**	ALL	2504	A: 17.17%, G: 82.83%
**African**	AFR	661	A: 17.02%, G: 82.98%
**Yoruba in Ibadan, Nigeria**	YRI	108	A: 12.96%, G: 87.04%
**Luhya in Webuye, Kenya**	LWK	99	A: 16.16%, G: 83.84%
**Gambian in Western Divisions in the Gambia**	GWD	113	A: 22.57%, G: 77.43%
**Mende in Sierra Leone**	MSL	85	A: 14.71%, G: 85.29%
**Esan in Nigeria**	ESN	99	A: 19.7%, G: 80.3%
**Americans of African Ancestry in SW USA**	ASW	61	A: 21.31%, G: 78.69%
**African Caribbeans in Barbados**	ACB	96	A: 12.5%, G: 87.5%
**Ad Mixed American**	AMR	347	A: 15.27%, G: 84.73%
**Mexican Ancestry from Los Angeles USA**	MXL	64	A: 14.84%, G: 85.16%
**Puerto Ricans from Puerto Rico**	PUR	104	A: 19.71%, G: 80.29%
**Colombians from Medellin, Colombia**	CLM	94	A: 20.21%, G: 79.79%
**Peruvians from Lima, Peru**	PEL	85	A: 4.71%, G: 95.29%
**East Asian**	EAS	504	A: 4.66%, G: 95.34%
**Han Chinese in Beijing, China**	CHB	103	A: 2.91%, G: 97.09%
**Japanese in Tokyo, Japan**	JPT	104	A: 3.37%, G: 96.63%
**Southern Han Chinese**	CHS	105	A: 1.9%, G: 98.1%
**Chinese Dai in Xishuangbanna, China**	CDX	93	A: 7.53%, G: 92.47%
**Kinh in Ho Chi Minh City, Vietnam**	KHV	99	A: 8.08%, G: 91.92%
**European**	EUR	503	A: 34.1%, G: 65.9%
**Utah Residents (CEPH) with Northern and Western European Ancestry**	CEU	99	A: 29.29%, G: 70.71%
**Toscani in Italia**	TSI	107	A: 32.71%, G: 67.29%
**Finnish in Finland**	FIN	99	A: 39.9%, G: 60.1%
**British in England and Scotland**	GBR	91	A: 31.87%, G: 68.13%
**Iberian Population in Spain**	IBS	107	A: 36.45%, G: 63.55%
**South Asian**	SAS	489	A: 14.21%, G: 85.79%
**Gujarati Indian from Houston, Texas**	GIH	103	A: 11.17%, G: 88.83%
**Punjabi from Lahore, Pakistan**	PJL	96	A: 14.06%, G: 85.94%
**Bengali from Bangladesh**	BEB	86	A: 11.05%, G: 88.95%
**Sri Lankan Tamil from the UK**	STU	102	A: 19.12%, G: 80.88%
**Indian Telugu from the UK**	ITU	102	A: 15.2%, G: 84.8%

Supplementary Table S7. Genotype distributions of rs6971 in individuals with METH-dependence and healthy controls.

		MA	MAP	HC	x^2	*P*
**Total (n=300)**	GG	62 (33.7%)	58 (31.5%)	64 (34.8%)	6.128	0.190
	GA	33 (35.5%)	29 (31.2%)	31 (33.3%)		
	AA	5 (21.7%)	13 (56.5%)	5 (21.7%)		
**Female (n=82)**	GG	7 (15.2%)	11 (23.9%)	28 (60.9%)	5.606	0.196*
	GA	6 (20.0%)	5 (16.7%)	19 (63.3%)		
	AA	-	4 (66.7%)	2 (33.3%)		
**Male (n=218)**	GG	55 (39.9%)	47 (34%)	36 (26.1%)	3.301	0.517
	GA	27 (42.9%)	24 (38.1%)	12 (19.0%)		
	AA	5 (29.4%)	9 (52.9%)	3 (17.7%)		

P values were calculated using the chi-square test. *Fisher's exact test was used. HC= Healthy Controls; MA= Methamphetamine addiction; MAP = Methamphetamine-associated psychosis.

Supplementary Table S8. Allele frequency distributions of *TSPO*/rs6971 in females.

	MA+MAP (n=33)	HC (n=49)	x^2	*P*
**Alleles**	G: 0.712 (71.2%)	G: 0.765 (76.5%)	0.586	0.444
	A: 0.288 (28.8%)	A: 0.235 (23.5%)		
**Genotypes**	GG (n=18)/GA (n=11)/AA (n=4)	GG (n=28)/GA (n=19)/AA (n=2)		
	**MA (n=13)**	**HC (n=49)**		
**Alleles**	G: 0.769 (76.9%)	G: 0.765 (76.5%)	0.002	0.967
	A: 0.231 (23.1%)	A: 0.235 (23.5%)		
**Genotypes**	GG (n=7)/GA (n=6)/AA (n=0)	GG (n=28)/GA (n=19)/AA (n=2)		
	**MAP (n=20)**	**HC (n=49)**		
**Alleles**	G: 0.675 (67.5%)	G: 0.765 (76.5%)	1.201	0.273
	A: 0.325 (32.5%)	A: 0.235 (23.5%)		
**Genotypes**	GG (n=11)/GA (n=5)/AA (n=4)	GG (n=28)/GA (n=19)/AA (n=2)		
	**MA (n=13)**	**MAP (n=20)**		
**Alleles**	G: 0.769 (76.9%)	G: 0.675 (67.5%)	0.683	0.409
	A: 0.231 (23.1%)	A: 0.325 (32.5%)		
**Genotypes**	GG (n=7)/GA (n=6)/AA (n=0)	GG (n=11)/GA (n=5)/AA (n=4)		

HC=Healthy Controls; MA= Methamphetamine addiction; MAP=Methamphetamine-associated psychosis**.** *P* values were calculated using the chi-square test.

Supplementary Table S9. Allele frequency distributions of *TSPO*/rs6971 in males.

	MA+MAP (n=167)	HC (n=51)	x^2	*P*
**Alleles**	G: 0.763 (76.3%)	G: 0.824 (82.4%)	1.629	0.202
	A: 0.237 (23.7%)	A: 0.176 (17.6%)		
**Genotypes**	GG (n=102)/GA (n=51)/AA (n=14)	GG (n=36)/GA (n=12)/AA (n=3)		
	**MA (n=87)**	**HC (n=51)**		
**Alleles**	G: 0.787 (78.7%)	G: 0.824 (82.4%)	0.527	0.468
	A: 0.213 (21.3%)	A: 0.176 (17.6%)		
**Genotypes**	GG (n=55)/GA (n=27)/AA (n=5)	GG (n=36)/GA (n=12)/AA (n=3)		
	**MAP (n=80)**	**HC (n=51)**		
**Alleles**	G: 0.737 (73.7%)	G: 0.824 (82.4%)	2.611	0.106
	A: 0.263 (26.3%)	A: 0.176 (17.6%)		
**Genotypes**	GG (n=47)/GA (n=24)/AA (n=9)	GG (n=36)/GA (n=12)/AA (n=3)		
	**MA (n=87)**	**MAP (n=80)**		
**Alleles**	G: 0.787 (78.7%)	G: 0.737 (73.7%)	1.147	0.284
	A: 0.213 (21.3%)	A: 0.263 (26.3%)		
**Genotypes**	GG (n=55)/GA (n=27)/AA (n=5)	GG (n=47)/GA (n=24)/AA (n=9)		

HC=Healthy Controls; MA= Methamphetamine addiction; MAP=Methamphetamine-associated psychosis. *P* values were calculated using the chi-square test.

Supplementary Table S10. Smoking-adjusted sensitivity logistic regression analyses of the association between *TSPO* rs6971 genotypes and MA/MAP status.

		MA+MAP/HC Total (n=300)					MA+MAP/HC Male (n=218)					MA+MAP/HC Female (n=82)				
		**MA+MAP**	**HC**	**β**	**OR (Cl 95 %)**	***P***	**MA+MAP**	**HC**	**β**	**OR (Cl 95 %)**	***P***	**MA+MAP**	**HC**	**β**	**OR (Cl 95 %)**	***P***
**Smoking (yes/no)**		198/2	44/56				166/1	27/24				32/1	17/32			
**Dominant**	AA+GA vs. GG	80-120	36-64	0.24	1.27 (0.64-2.49)	0.495	65-102	15-36	0.20	1.22 (0.53-2.82)	0.641	15-18	21-28	0.28	1.33 (0.42-4.19)	0.629
**Recessive**	AA vs. GA+GG	18-182	5-95	0.34	1.40 (0.40-4.92)	0.599	14-153	3-48	0.11	1.12 (0.25-5.07)	0.883	4-29	2-47	0.75	2.11 (0.24-18.27)	0.497
**Co-dominant**	GG	120	64	-	-	*Ref*	102	36	-	-	*Ref*	18	28	-	-	*Ref*
	GA vs. GG	62-120	31-64	0.19	1.21 (0.59-2.51)	0.604	51-102	12-36	0.21	1.23 (0.49-3.06)	0.658	11-18	19-28	0.15	1.16 (0.34-3.97)	0.810
	AA vs. GG	18-120	5-64	0.40	1.49 (0.42-5.35)	0.539	14-102	3-36	0.18	1.19 (0.26-5.53)	0.821	4-18	2-28	0.80	2.23 (0.25-20.21)	0.476
**Overdominant**	GA vs. GG+AA	62-138	31-69	0.15	1.16 (0.57-2.37)	0.688	51-116	12-39	0.19	1.21 (0.49-2.96)	0.684	11-22	19-30	0.05	1.05 (0.32-3.48)	0.938
**Additive**	-	-	-	0.20	1.22 (0.72-2.05)	0.459	-	-	0.14	1.15 (0.60-2.18)	0.677	-	-	0.30	1.35 (0.56-3.24)	0.508
		**MA/HC Total (n=200)**					**MA/HC Male (n=138)**					**MA/HC Female (n=62)**				
		**MA**	**HC**	**β**	**OR (Cl 95 %)**	***P***	**MA**	**HC**	**β**	**OR (Cl 95 %)**	***P***	**MA**	**HC**	**β**	**OR (Cl 95 %)**	***P***
**Smoking (yes/no)**		99/1	44/56				87/0	27/24				12/1	17/32			
**Dominant**	AA+GA vs. GG	38-62	36-64	0.21	1.23 (0.58-2.64)	0.590	32-55	15-36	NE	NE	-	6-7	21-28	0.30	1.35 (0.33-5.47)	0.678
**Recessive**	AA vs. GA+GG	5-95	5-95	-0.49	0.61 (0.14-2.78)	0.527	5-82	3-48	NE	NE	-	0-13	2-47	NE	NE	-
**Co-dominant**	GG	62	64	-	-	*Ref*	55	36	-	-	*Ref*	7	28	-	-	*Ref*
	GA vs. GG	33-62	31-64	0.33	1.39 (0.61-3.14)	0.435	27-55	12-36	NE	NE	-	6-7	19-28	NE	NE	-
	AA vs. GG	5-62	5-64	-0.39	0.68 (0.15-3.15)	0.622	5-55	3-36	NE	NE	-	0-7	2-28	NE	NE	-
**Overdominant**	GA vs. GG+AA	33-67	31-69	0.36	1.43 (0.64-3.20)	0.386	27-60	12-39	NE	NE	-	6-7	19-30	0.54	1.71 (0.41-7.11)	0.459
**Additive**	-	-	-	0.06	1.06 (0.57-1.97)	0.845	-	-	NE	NE	-	-	-	0.05	1.06 (0.31-3.61)	0.932
		**MAP/HC Total (n=200)**					**MAP/HC Male (n=131)**					**MAP/HC Female (n=69)**				
		**MAP**	**HC**	**β**	**OR (Cl 95 %)**	***P***	**MAP**	**HC**	**β**	**OR (Cl 95 %)**	***P***	**MAP**	**HC**	**β**	**OR (Cl 95 %)**	***P***
**Smoking (yes/no)**		99/1	44/56				79/1	27/24				20/0	17/32			
**Dominant**	AA+GA vs. GG	42-58	36-64	0.15	1.17 (0.55-2.47)	0.691	33-47	15-36	0.19	1.21 (0.49-3.03)	0.679	9-11	21-28	NE	NE	-
**Recessive**	AA vs. GA+GG	13-87	5-95	0.74	2.09 (0.56-7.79)	0.274	9-71	3-48	0.39	1.48 (0.31-7.05)	0.627	4-16	2-47	NE	NE	-
**Co-dominant**	GG	58	64	-	-	*Ref*	47	36	-	-	*Ref*	11	28	-	-	*Ref*
	GA vs. GG	29-58	31-64	-0.04	0.97 (0.43-2.19)	0.931	24-47	12-36	0.12	1.12 (0.41-3.06)	0.820	5-11	19-28	NE	NE	-
	AA vs. GG	13-58	5-64	0.72	2.06 (0.54-7.92)	0.293	9-47	3-36	0.43	1.53 (0.31-7.56)	0.601	4-11	2-28	NE	NE	-
**Overdominant**	GA vs. GG+AA	29-71	31-69	-0.14	0.87 (0.39-1.93)	0.730	24-56	12-39	0.06	1.06 (0.40-2.82)	0.909	5-15	19-30	NE	NE	-
**Additive**	-	-	-	0.22	1.25 (0.72-2.16)	0.429	-	-	0.18	1.20 (0.61-2.35)	0.603	-	-	NE	NE	-

In the dominant and co-dominant models, the GG genotype was used as the reference group; for the recessive model, the GA+GG genotypes were used as the reference; and for the overdominant model, the reference group was GG+AA. Male was selected as the reference group for gender. Age, gender, and smoking status were included as covariates in the relevant models. Benjamini–Hochberg FDR correction was applied, and all q values were >0.05. β: Beta coefficient; OR: Odds ratio; CI: Confidence interval; n: Number of individuals. Statistically significant results are presented in bold. HC=Healthy controls; MA= Methamphetamine addiction; MAP=Methamphetamine-associated psychosis. NE, not estimable because adjustment for smoking resulted in complete or quasi-complete separation and model non-convergence. Smoking-adjusted associations were assessed using binary logistic regression.

Supplementary Table S11. Distribution of *TSPO*/rs6971 genotypes in *TSPO* (+) and *TSPO* (-) samples determined by qRT-PCR.

	**MA (n=29)**	**MAP (n=33)**	**MA+MAP (n=62)**	**HC (n=28)**
	+/-, n (%)	+/-, n (%)	+/-, n (%)	+/-, n (%)
**GG**	0/10, 10 (34.48)	2/9, 11 (33.33)	2/19, 21 (33.87)	8/3, 11 (39.29)
**GA**	6/8, 14 (48.28)	3/8, 11 (33.33)	9/16, 25 (40.32)	10/2, 12 (42.85)
**AA**	1/4, 5 (17.24)	2/9, 11 (33.33)	3/13, 16 (25.81)	5/0, 5 (17.85)
**GG vs. GA+AA**	***P*=0.032***	*P*=0.571*	*P*=0.071*	*P*=0.290*
**GA vs. GG+AA**	***P*=0.031***	*P*=0.429*	***P*=0.039***	*P*=0.671*

*Fisher's exact test was used. HC= Healthy Controls; MA= Methamphetamine addiction; MAP= Methamphetamine-associated psychosis

Supplementary Table S12. Comparison of TSPO protein levels in the study groups stratified by genotype.

**rs6971**	**MA+MAP n (%)**	**HC n (%)**	**MA n (%)**	**MAP n (%)**	**MA+MAP mean±SD**	**HC mean±SD**	**MA mean±SD**	**MAP mean±SD**	***P₁***	***P₂***	***P₃***	***P₄***
GG	21 (34.99)	11 (39.28)	10 (35.71)	11 (34.38)	0.481±0.159	0.671±0.372	0.496±0.177	0.467±0.148	0.069	0.137	0.112	0.987
GA	23 (38.33)	12 (42.85)	13 (46.42)	10 (31.25)	0.739±0.501	0.630±0.258	0.549±0.260	0.986±0.636	0.898	0.264	0.283	**0.036**
AA	16 (26.66)	5 (17.85)	5 (17.85)	11 (34.38)	0.788±0.514	0.499±0.294	0.764±0.484	0.799±0.549	0.094	0.095	0.189	0.661
P/x²		0.494/3.40			*P*=0.080	*P*=0.129	*P*=0.382	***P*=0.043**				

P_1: Difference between the combined MA+MAP group and healthy controls; ***P_2***: Difference between the MA group and healthy controls; ***P_3***: Difference between the MAP group and healthy controls; ***P_4***: Difference between the MA and MAP groups. Statistically significant results are presented in bold. HC= Healthy controls; MA= Methamphetamine addiction; MAP= Methamphetamine-associated psychosis

Supplementary Table S13. Functional relevance of rs6971 retrieved from RegulomeDB and GTEx databases.

RegulomeDB Score	Gencode ID	Gene Symbol	*P*-Value	NES	Tissue
1f[a]	ENSG00000100290.3	*BIK*	0.0000058	-0.11	Whole Blood
	ENSG00000274717.1	*ENSG00000274717*	0.00012	0.16	Thyroid
	ENSG00000100300.18	*TSPO*	5.7e-18	0.18	Cells - Cultured fibroblasts
	ENSG00000100300.18	*TSPO*	2.5e-17	0.21	Adipose - Subcutaneous
	ENSG00000100300.18	*TSPO*	1.2e-12	0.078	Whole Blood
	ENSG00000100300.18	*TSPO*	1.3e-8	0.13	Lung
	ENSG00000100300.18	*TSPO*	1.7e-8	-0.18	Testis
	ENSG00000100300.18	*TSPO*	0.0000016	0.20	Brain - Substantia nigra
	ENSG00000100300.18	*TSPO*	0.0000022	0.12	Nerve - Tibial
	ENSG00000100300.18	*TSPO*	0.0000029	0.11	Breast - Mammary Tissue
	ENSG00000100300.18	*TSPO*	0.000021	0.11	Adipose - Visceral (Omentum)
	ENSG00000100300.18	*TSPO*	0.000033	-0.27	Cells - EBV-transformed lymphocytes
	ENSG00000100304.13	*TTLL12*	5.3e-7	-0.16	Esophagus - Muscularis
	ENSG00000100304.13	*TTLL12*	6.2e-7	-0.11	Skin - Sun Exposed (Lower leg)
	ENSG00000100304.13	*TTLL12*	0.0000022	-0.15	Artery - Tibial

a;1f; eQTL/caQTL + TF binding / chromatin accessibility peak, NES: Normalized Effect Score

SUPPLEMENTARY FIGURES

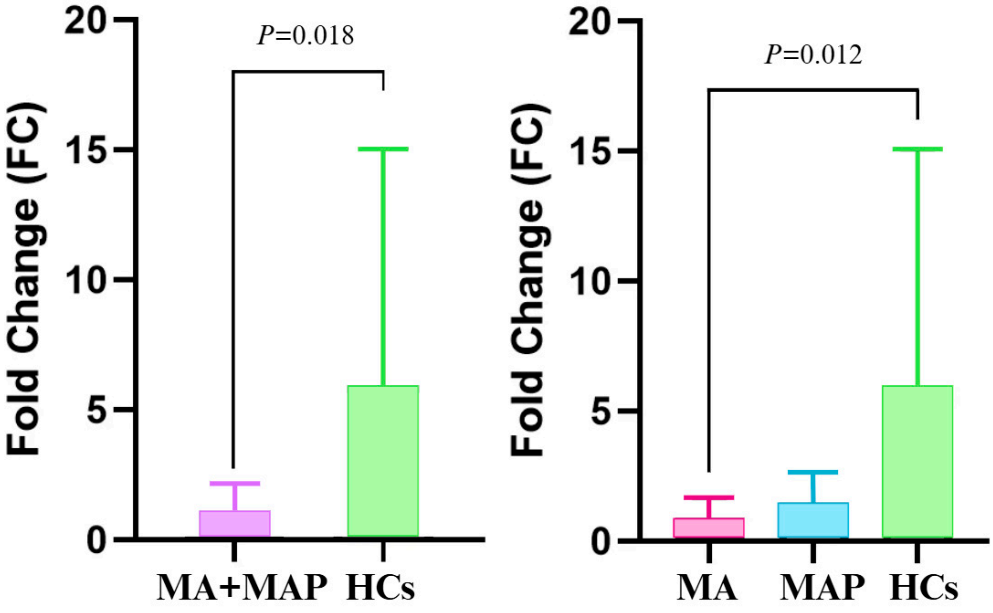

Supplementary Figure S1. Analysis of relative *TSPO* expression levels following the exclusion of samples assigned a Ct value of 40. The Mann–Whitney U test was used for the two-group comparison, and the Kruskal–Wallis test with Dunn's post hoc test was used for the three-group comparison.
